# Functional genetic characterization of salivary gland development in *Aedes aegypti*

**DOI:** 10.1186/2041-9139-4-9

**Published:** 2013-03-06

**Authors:** Chilinh Nguyen, Emily Andrews, Christy Le, Longhua Sun, Zeinab Annan, Anthony Clemons, David W Severson, Molly Duman-Scheel

**Affiliations:** 1 University of Notre Dame, Notre Dame, Eck Institute for Global Health and Department of Biological Sciences, Notre Dame, IN 46556, USA; 2 Department of Medical and Molecular Genetics, Indiana University School of Medicine, South Bend, IN 46617, USA; 3 Indiana University School of Medicine, Raclin-Carmichael Hall, 1234 Notre Dame Avenue, South Bend, IN 46617, USA

**Keywords:** *Aedes aegypti*, CrebA, Development, *Drosophila melanogaster*, Mosquito, Salivary gland, siRNA, Vector

## Abstract

**Background:**

Despite the devastating global impact of mosquito-borne illnesses on human health, very little is known about mosquito developmental biology. In this investigation, functional genetic analysis of embryonic salivary gland development was performed in *Aedes aegypti*, the dengue and yellow fever vector and an emerging model for vector mosquito development. Although embryonic salivary gland development has been well studied in *Drosophila melanogaster*, little is known about this process in mosquitoes or other arthropods.

**Results:**

Mosquitoes possess orthologs of many genes that regulate *Drosophila melanogaster* embryonic salivary gland development. The expression patterns of a large subset of these genes were assessed during *Ae. aegypti* development. These studies identified a set of molecular genetic markers for the developing mosquito salivary gland. Analysis of marker expression allowed for tracking of the progression of *Ae. aegypti* salivary gland development in embryos. In *Drosophila,* the salivary glands develop from placodes located in the ventral neuroectoderm. However, in *Ae. aegypti,* salivary marker genes are not expressed in placode-like patterns in the ventral neuroectoderm. Instead, marker gene expression is detected in salivary gland rudiments adjacent to the proventriculus. These observations highlighted the need for functional genetic characterization of mosquito salivary gland development. An siRNA- mediated knockdown strategy was therefore employed to investigate the role of one of the marker genes, *cyclic-AMP response element binding protein A* (*Aae crebA*)*,* during *Ae. aegypti* salivary gland development*.* These experiments revealed that *Aae crebA* encodes a key transcriptional regulator of the secretory pathway in the developing *Ae. aegypti* salivary gland.

**Conclusions:**

The results of this investigation indicated that the initiation of salivary gland development in *Ae. aegypti* significantly differs from that of *D. melanogaster*. Despite these differences, some elements of salivary gland development, including the ability of CrebA to regulate secretory gene expression, are conserved between the two species. These studies underscore the need for further analysis of mosquito developmental genetics and may foster comparative studies of salivary gland development in additional insect species.

## Background

Despite the devastating global impact of mosquito-borne illnesses on human health, vector mosquito developmental biology has been grossly understudied. Although excellent descriptions of the development of some species, including the dengue and yellow fever vector mosquito *Aedes aegypti* have been compiled, detailed expression patterns of only a handful of mosquito embryonic genes have been described in this or any other mosquito species [1-3]. We recently began to address the need for developmental research in mosquitoes through the pursuit of functional developmental genetic studies in *Ae. aegypti*[4-10]. A number of key advantages make *Ae. aegypti* an excellent species in which to study mosquito developmental biology. Females of this species lay eggs on artificial substrates such as paper towels, which facilitates the narrowly timed egg collections that are often required for developmental studies. Also, *Ae. aegypti* undergo egg diapause, an advantage that permits egg storage and which significantly decreases the amount of labor required for maintenance of transgenic strains. Furthermore, our recent survey suggests that orthologs of many developmental genes are found in the *Ae. aegypti* genome [11]. We recently published methodology for functional analysis of developmental genes in *Ae. aegypti*. These methods, including procedures for egg collection, tissue preparation, gene and protein expression, and knockdown of developmental genes [4-8], in conjunction with previously published mosquito protocols [12,13] are facilitating functional developmental genetic studies in *Ae. aegypti.* It is anticipated that the analysis of development in *Ae. aegypti*, an emerging model for vector mosquito development, will foster comparative studies in other vector mosquito species. Here, we investigate development of the *Ae. aegypti* salivary gland.

The salivary glands of blood-feeding adult female vector mosquitoes have been the subject of many research investigations. The saliva secreted by hematophagous mosquitoes contains antihemostatic and anti-inflammatory agents. These secretions counteract host responses that might restrict blood flow or draw attention to the feeding site and are critical for blood-feeding behavior in adult female mosquitoes [14,15]. The salivary glands of adult females are also crucial for pathogen transmission to human hosts. Following salivary gland infection, female mosquitoes are competent for disease transmission for the duration of their lives [14]. Although adult salivary glands have been intensely studied, genetic analysis of the developing embryonic mosquito salivary gland, the focus of this investigation, has not been pursued. Moreover, analysis of mosquito salivary gland development is of significant importance to the comparative arthropod development community, as we presently lack knowledge of arthropod salivary gland developmental genetics outside of *Drosophila melanogaster,* a genetic model in which it has been studied extensively [16,17].

In *D. melanogaster*, salivary gland development initiates from two primordia, the salivary gland placodes, that are located in the ventral neuroectoderm (VNE). During development, cells from each individual placode invaginate, forming internalized pairs of secretory tubes that elongate dorsally [16,17]. It was hypothesized at the onset of our investigation that salivary gland development in other dipteran insects would resemble that of *D. melanogaster.* However, recent studies that uncovered differences between *D. melanogaster* and mosquito embryonic development highlighted the critical importance of experimentally testing this hypothesis [9-11]. Furthermore, although mosquito salivary gland development has not been assessed genetically, a morphological description of the development of the inland freshwater mosquito *Ae. vexans*[18] suggested that the salivary glands of this aedine mosquito species develop as outpocketings of the proventriculus, the intussusception of the end of the esophagus into the midgut [19]. This brief description suggested that the origins of the mosquito salivary gland may differ from *D. melanogaster*[16,17] and further emphasized the need to experimentally test our hypothesis.

In this investigation, we identified genetic markers for the developing *Ae. aegypti* embryonic salivary gland which were used to track the origins and progression of the development of this tissue. Analysis of marker gene expression revealed significant differences between *Ae. aegypti* and *D. melanogaster* salivary gland development, and these differences underscored the need for thorough functional genetic characterization of the development of this tissue in mosquitoes. Here, we initiated these studies by employing an siRNA-mediated knockdown strategy to investigate the role of one of the marker genes, *cyclic-AMP response element binding protein A (Aae crebA),* during *Ae. aegypti* salivary gland development.

## Methods

### Mosquito rearing, egg collection and fixation

The *Ae. aegypti* Liverpool-IB12 (LVP-1B12) strain was used in these studies. Procedures for mosquito rearing, egg collection [4] and fixation [5] have been previously described. Blood feeding of mosquitoes was performed in accordance with the recommendations in the Guide for the Care and Use of Laboratory Animals of the National Institutes of Health and an animal use protocol that was approved by the University of Notre Dame Institutional Animal Care and Use Committee (Study # 14–026).

### Immunohistochemistry

Immunohistochemistry was performed as described previously [20]. Anti-*Drosophila* CrebA was obtained from the Developmental Studies Hybridoma Bank (University of Iowa, Iowa City, IA, USA), and horseradish peroxidase-conjugated secondary antibodies were purchased from Jackson Immunoresearch (West Grove, PA, USA).

### Sequence analyses

Candidate marker genes were selected on the basis of their orthology to known salivary gland markers in *D. melanogaster,* which were selected through literature searches and by using the ontology search tool in FlyExpress [21,22]. Orthology calls were prepared with the aid of Biomart [23] and VectorBase [24]. Genes with 1:1 orthology to the relevant *D. melanogaster* genes were chosen for expression analyses. cAMP response element (CRE) sites upstream of these genes were identified through assessment of the 5′ flanking regions, which were exported from VectorBase and searched for sequences corresponding to the CRE consensus sequence [25].

### *In situ* hybridization

Riboprobes corresponding to the *Ae. aegypti* genes listed in Additional file 1 were synthesized according to the Patel protocol [26]. *In situ* hybridization experiments were performed as previously described [8].

### RNAi experiments

Knockdown of *Aae crebA* was performed through embryonic microinjection of siRNAs corresponding to this gene. siRNA design and microinjection were performed using previously published methodology [7], which has been utilized in two recent knockdown investigations [9,10]. The following siRNAs corresponding to *Ae. aegypti crebA* were synthesized by Dharmacon RNAi Technologies (Lafayette, CO, USA): siRNA-1 sense, GCAGUCAACCG GUAAGAUA and antisense, CGUCAGUUGGCCAUUC UAU (corresponds to base pairs of 816 to 834 of *Aae crebA*); and siRNA-2 sense, UGUUGGAGCACAAGGU AGU and antisense, ACAACCUCGUGUUCCAUCA (corresponds to base pairs 47 to 488 of *Aae crebA*). A scrambled version of siRNA-1 was used as a control: sense, GAAACGCACGUAAGUAGUC and antisense, CUUUGC GUGCAUUCAUCAG. All siRNAs were injected at a concentration of 6 μg/uL. In all cases, two to three replicate experiments (n = 30 per control or experimental group) were performed with both siRNA-1 and siRNA-2. Measurement of knockdown effectiveness was assessed through *in situ* hybridization.

## Results

### The initial stages of salivary gland development in *Ae. aegypti* and *D. melanogaster* differ

**Figure 1:**
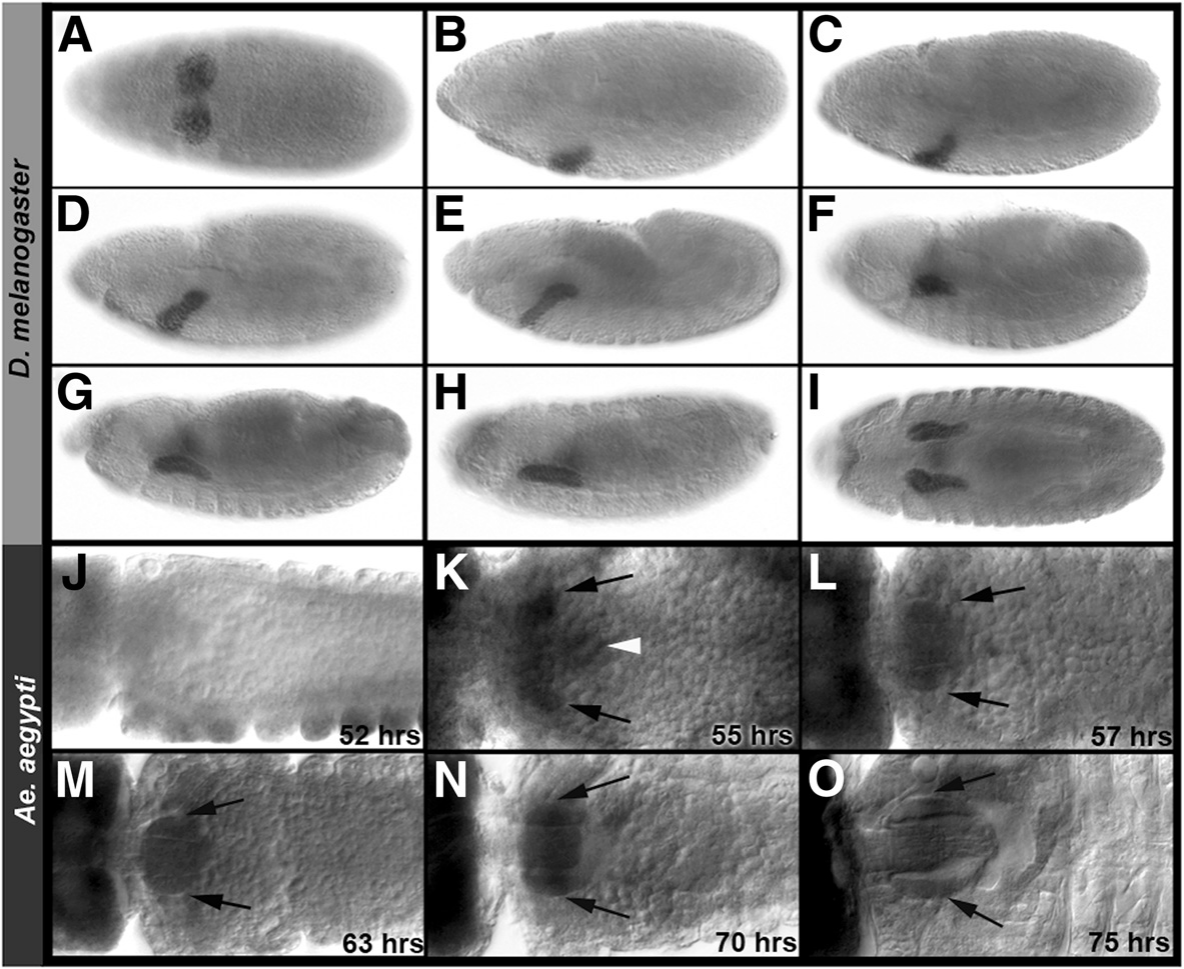
**Initiation of salivary gland development in *Ae. aegypti *and *D. melanogaster *differs.** (**A**-**I**) CrebA protein expression marks the developing *D. melanogaster* salivary gland in lateral views of progressively older *D. melanogaster* embryos. Stages 9 (**A**) through 15 (**I**) are shown. (**A**) The *D. melanogaster* salivary gland initiates from two salivary gland placodes located in the VNE. (**B**-**H**) During development, cells from each individual placode invaginate forming internalized pairs of secretory tubes that elongate dorsally. (**I**) A ventral view of both glands in a Stage 15 embryo is shown. (**J**) In *Ae. aegypti,* expression of *crebA* mRNA transcript is not detected in VNE placodes. (**K**) Instead, salivary gland rudiments marked by *crebA* RNA expression (arrows, 55-h embryo) form adjacent to the proventriculus (white arrowhead). (**L**) By 57 h, secretory tubes (arrows) have formed. (**M**, **N**) These enlarge as development progresses. (**O**) Prominent thoracic structures located on either side of the proventriculus are evident at 75 h of development (arrows). (**K**-**O**) Dorsal views are shown. Embryos are oriented anterior-left in all panels.

**Figure 2:**
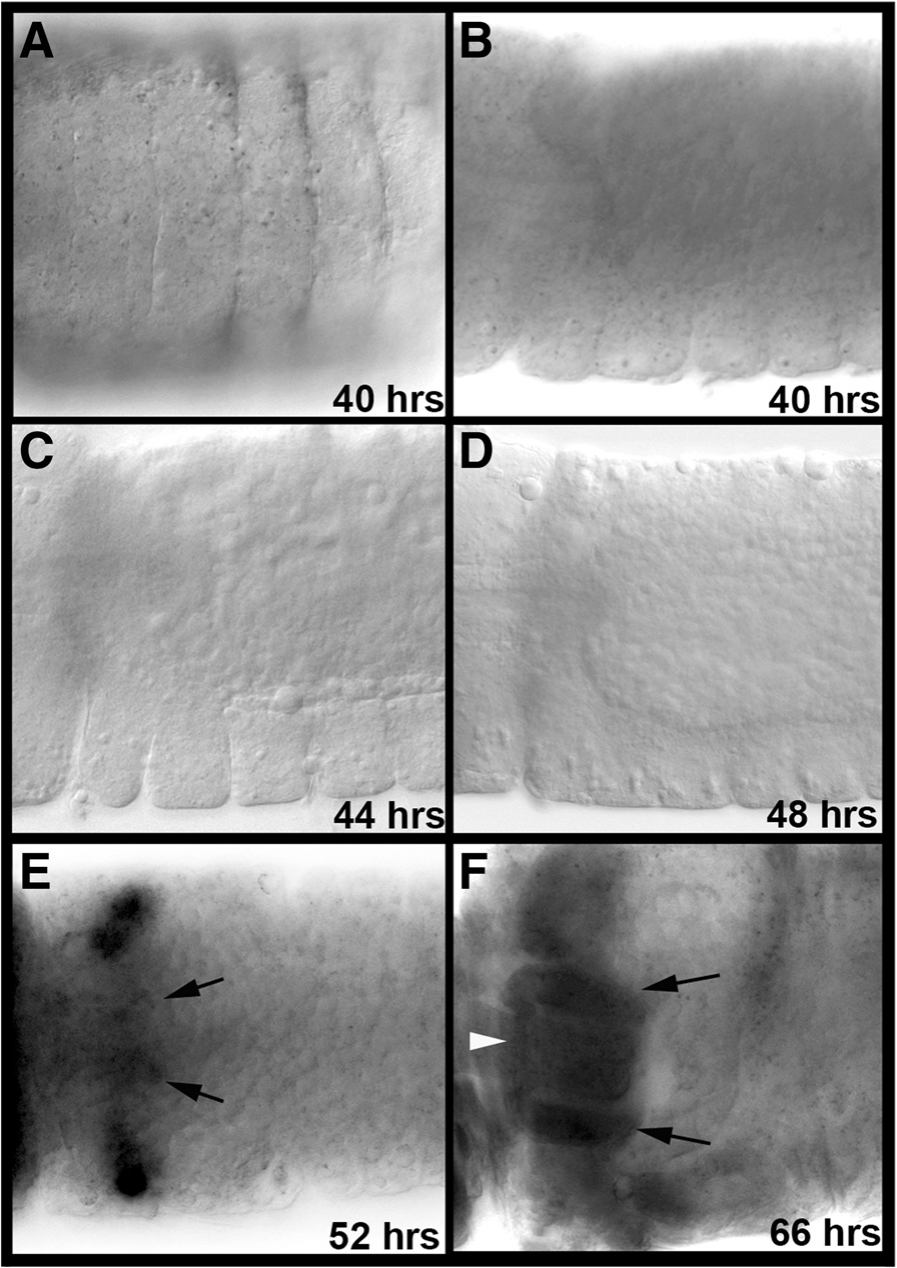
***Aae paps *expression is detected in salivary glands developing adjacent to the proventriculus.** (**A**-**D**) *Aae paps* expression is not detected in placodes in the VNE (ventral view of anterior segments in (**A**), lateral views of anterior segments in (**B-D**); respective ages are indicated). (**E**) *Aae paps* expression is detected in the developing salivary glands on either side of the proventriculus at 52 h of development (dorsal view shown). (**F**) At 66 h of development, large salivary tubes with high levels of *Aae paps* expression are evident (arrows, dorsal view is shown). The two lateral ducts attached to the anterior portion of the secretory tubes express *paps* and have joined (white arrowhead). Embryos are oriented anterior-left in all panels.

A number of *D. melanogaster* salivary gland marker genes were selected through literature and FlyExpress ontology [21,22] searches. Expression of the mosquito orthologs of these genes (Additional file 1), which were predicted to function as mosquito salivary gland markers, was assessed during *Ae. aegypti* embryogenesis. At the onset of these studies, RNA expression patterns of *crebA* and *PAPS synthetase* (*paps*), which function as both early and late markers of the developing *D. melanogaster* salivary gland [16,21,22], were analyzed in detail at multiple embryonic stages of *Ae. aegypti* development. Although expression of both of these genes marks the salivary gland placodes of the fruit fly, *Aae crebA* and *Aae paps* expression was not detected in VNE placode-like patterns (Figures 1J and 2A-D; compare with *Drosophila* embryos in Figure 1A,B). Furthermore, thorough morphological analyses in concert with these gene expression studies provided no indication that invagination of cells from the VNE gives rise to tubal salivary gland structures in this species. These results suggested that the origins of the *Ae. aegypti* salivary glands may not reside in VNE placodes as they do in *D. melanogaster.*

In light of these findings, beginning with first instar larvae in which the salivary glands are prominent structures located adjacent to the proventriculus [19], the origin of the larval salivary glands was investigated through examination of progressively earlier stages of development. These studies, which were performed in conjunction with analysis of the expression patterns of *Aae crebA* and *paps,* demonstrated that the salivary glands develop from two rudiments located on either side of the proventriculus, where expression of both genes (*Aae crebA* in Figure 1K and *Aae paps* in Figure 2E) could be detected beginning at 50 hours of development. Collectively, these results suggested that the initial stages of *Ae. aegypti* embryonic salivary gland development significantly differ from that of *D. melanogaster.*

### Embryonic development of the *Ae. aegypti* salivary gland

Analysis of *Aae crebA* and *paps* expression from 50 hours of development through later stages of embryogenesis allowed for tracking of the progression of salivary gland development, which was assessed in greater detail. The salivary gland rudiments consist of two different cell types: preduct and presecretory cells. During embryogenesis, the presecretory cells form simple tubular glands (Figure 1L) that elongate as embryogenesis progresses (Figure 1M-O). By 75 hours of development, the secretory tubes are prominent thoracic structures located on either side of the proventriculus (Figure 1O), and this position is retained through hatching.

In addition to the secretory tubes, *paps* expression serves as a marker for the developing lateral ducts, which also arise from the salivary gland rudiment (Figure 2E,F). The two lateral ducts, each of which is attached to the anterior end of the developing secretory tube located on the same side, join with each other during embryogenesis (Figure 2F). Later, during the larval stages, a central common duct (which is not visible in embryos) will connect the lateral ducts to the larval mouth. Together, the larval ducts form a Y-shaped tube through which secretions of the gland are transported to the larval mouth [19].

### *Ae. aegypti* salivary gland markers include secretory pathway component genes

**Figure 3:**
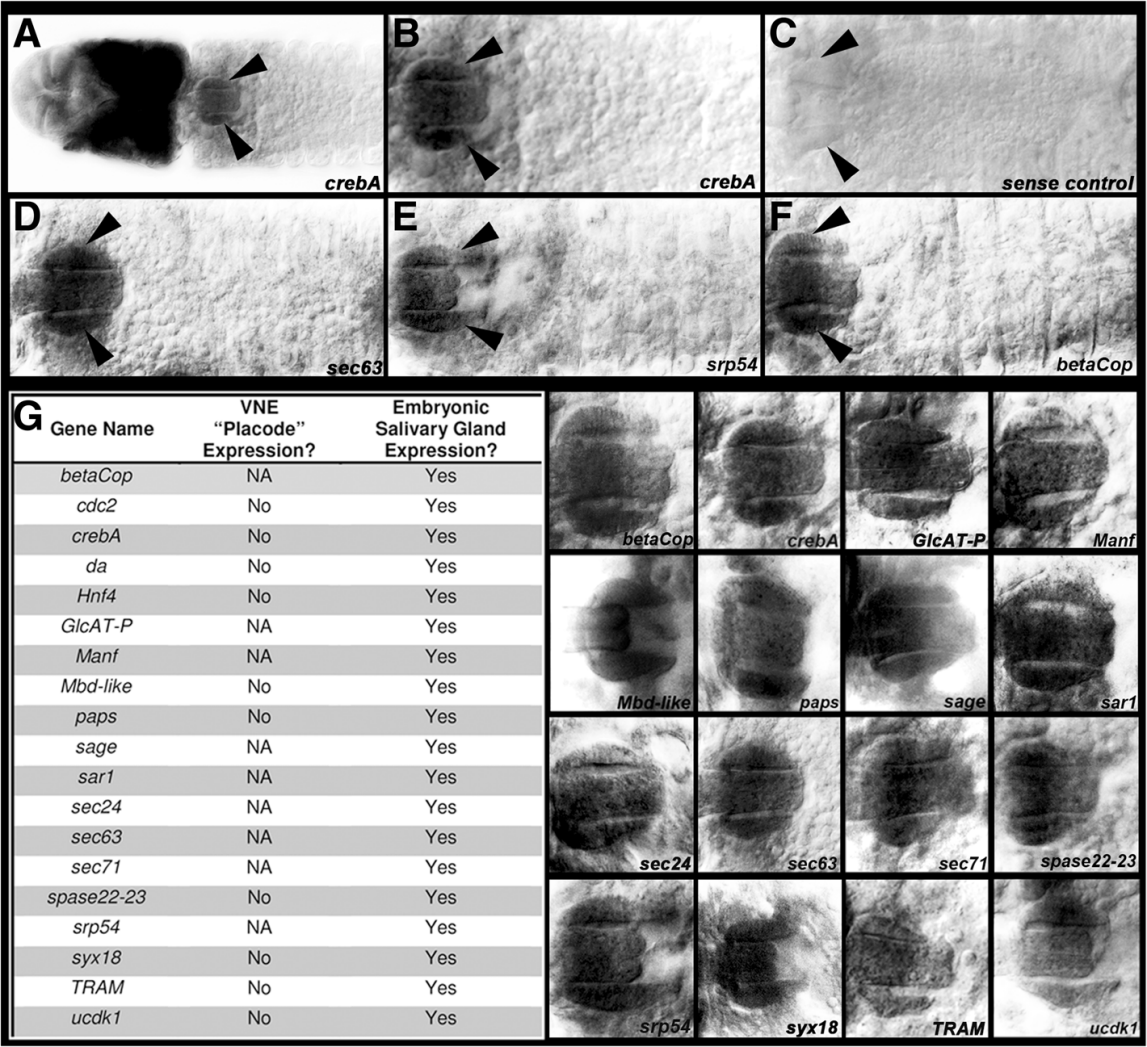
***Ae. aegypti *salivary gland markers.** Expression of *crebA* marks the developing salivary glands (arrowheads) in *Ae. aegypti*. (**A**) shows a low magnification view of the head and anterior segments of a 68-h embryo. (**B**) A higher magnification view of the salivary glands of this embryo is shown. (**C**) shows a negative sense control probe stain. (**D**-**G**) Expression of (**D**) *sec63*, (**E**) *srp54*, (**F**) *betaCop* and (**G**) a number of additional marker genes also labels the developing *Ae. aegypti* salivary glands. (**C**-**F**) display regions comparable to that which is shown in (**B**). (**G**) High magnification views of only the salivary glands are shown at right*.* Although expression of all the genes listed in (**G**) was detected in the developing embryonic salivary gland (signified by ‘Yes’ at left), *Drosophila* VNE placode-like expression patterns of these genes were not observed at the relevant stages of embryonic development in *Ae. aegypti* (signified by ‘No’ at left; NA indicates that gene expression was not assayed at the onset of salivary gland development). In all panels, marker gene expression is shown in dorsal views of *Ae. aegypti* salivary glands that are oriented anterior-left (embryos range in age from 65 to 70 h).

Probes corresponding to a number of additional *Ae. aegypti* orthologs of known *Drosophila* salivary gland marker genes were synthesized. A full list of these genes, including their putative functions and their orthology relationships with respect to the *Drosophila* genes, is provided in Additional file 1. Analysis of the expression of these additional markers, including mosquito orthologs of several genes that are expressed in the *D. melanogaster* VNE placodes (*sage*, *sec63*, *syx18* and *TRAM*) [21,22], provided further confirmation that the *Ae. aegypti* salivary gland rudiments do not reside in the VNE. Despite these observed differences between the initiation of *Ae. aegypti* and *D. melanogaster* development, each gene examined was found to be expressed in the developing *Ae. aegypti* salivary glands adjacent to the proventriculus (Figure 3). It should be noted that while these genes are referred to as salivary gland markers, their expression, like that of their *D. melanogaster* counterparts, can often be detected in multiple *Ae. aegypti* embryonic tissues. For example, *Aae crebA* expression marks the developing salivary gland but is also abundant throughout the embryonic head (Figure 3A).

A number of the marker genes identified encode members of the early secretory pathway. These genes, referred to as the secretory pathway component genes (SPCGs), encode the protein machinery that target and translocate proteins in the endoplasmic reticulum, cleave the N-terminal signal sequence, and regulate vesicle transport between the endoplasmic reticulum and Golgi. SPCG genes function in every cell, but are upregulated in the salivary gland given the secretory function of this organ [25]. A list of identified *Ae. aegypti* SPCG gene orthologs, including their putative functions and orthology relationships with respect to the *Drosophila* genes, is provided in Additional file 2. Expression of a subset of these genes was assessed in the developing *Ae. aegypti* salivary gland: *srp54*, *sec63*, *sec71*, *TRAM*, *spase22-23*, *sar1* and *sec24* (Figure 3D,E,G; see Additional file 1 for more information about each gene). As expected, expression of these *Aae* SPCG orthologs was found to be enriched in the developing salivary glands.

### CrebA is a regulator of secretory pathway genes in *Ae. aegypti*

**Figure 4:**
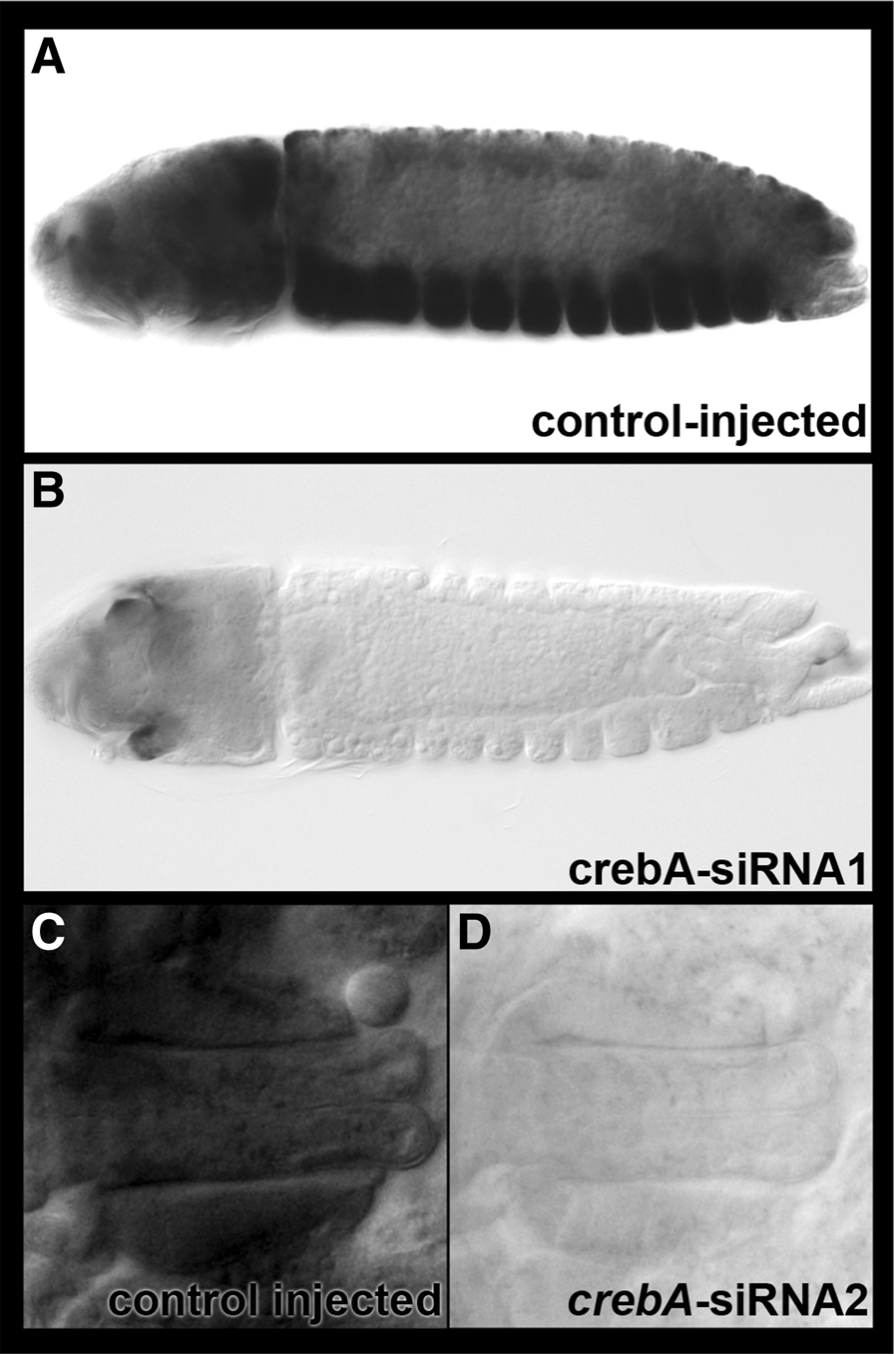
**siRNA-mediated knockdown of *crebA*.***Aae crebA* knockdown was confirmed through whole-mount *in situ* hybridization. (**A**, **B**) Lateral views of 54-h embryos. (**C**, **D**) Dorsal views of 68-h embryos. Two siRNAs targeting *crebA,* siRNA-1 (**B**) and siRNA-2 (**D**), resulted in loss of *crebA* levels throughout the entire embryo (**B**), including the salivary glands (**D**). Note that the darkened area in the head shown in (**B**) is normal pigmentation, not gene expression. *crebA* levels were not altered by injection of a scrambled control version of siRNA-1 (**A**, **C**). All panels are oriented anterior-left.

CrebA is a transcriptional activator of several *Drosophila* SPCGs [25]. CRE sequences, the sites to which CrebA binds, were identified in the 5′ upstream enhancer regions of a number of *Ae. aegypti* SPCGs (Additional file 2), suggesting that CrebA might regulate expression of these genes. This hypothesis was functionally tested through use of siRNA-mediated knockdown, which was recently shown to be an effective method of inhibiting gene expression during embryonic development of *Ae. aegypti*[9,10]. Experimental or control siRNAs were injected at precellular blastoderm. Two siRNAs corresponding to different regions of the *Aae crebA* gene, *crebA* siRNA-1 and *crebA* siRNA-2, were used to target this gene. Control experiments were performed using a scrambled control version of siRNA-1, which did not bear high sequence homology to other genes in the *Ae. aegypti* genome and did not impact *crebA* levels postinjection (Figure 4A,C). *Aae crebA* knockdown was confirmed through whole-mount *in situ* hybridization, which showed that injection of either *crebA* siRNA-1 or siRNA-2 significantly reduced *crebA* transcript levels, resulting in nearly complete loss of *crebA* transcripts throughout the embryo, including the developing salivary gland (Figure 4B,D). This knockdown persisted at least through late embryogenesis.

**Figure 5:**
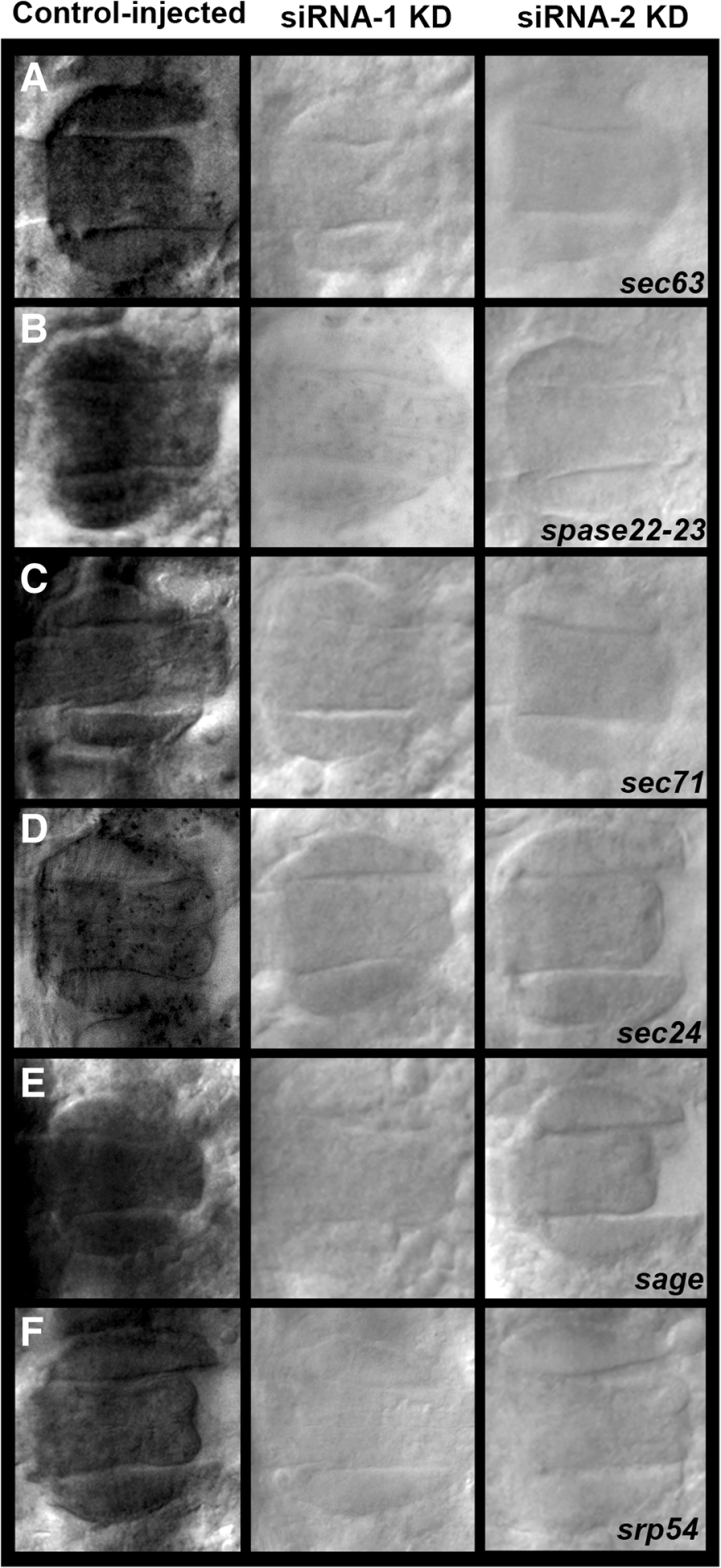
**Knockdown of *crebA *inhibits expression of secretory genes.** (**A**-**F**) Injection of siRNAs targeting *crebA* (siRNA-1, center; siRNA-2, right) resulted in significantly reduced transcript levels of secretory genes in the embryonic salivary gland, including: (**A**) *sec63*, (**B**) *spase22-23*, (**C**) *sec 71*, (**D**) *sec 24*, (**E**) *sage*, (**F**) *srp54*. Injection of scrambled control siRNA-1 (left) did not impact levels of any of the transcripts examined. Dorsal views oriented anterior left are shown in all figures. Embryos range in age from 65 to 70 h. These results identify CrebA as a key regulator of *Ae. aegypti* secretory genes in the developing salivary gland.

The impact of *crebA* knockdown on a subset of *Ae. aegypti* SPCGs was assessed. Injection of siRNA-1 was found to block salivary gland expression of all genes examined, which included: *sec24*, *sec63*, *sec71*, *spase22-23* and *srp54*, as well as the non-SPCG *sage* (Figure 5A-F, center). Comparable results were obtained when siRNA-2 was injected (Figure 5A-F, right), suggesting that the knockdown phenotypes observed were not the result of offsite targeting effects. Injection of scrambled control siRNA-1 did not impact the levels of any of these SPCGs (Figure 5A-F, right). These results identify CrebA as a key regulator of secretory gene activity in *Ae. aegypti.*

## Discussion

### Divergent and conserved mechanisms of insect salivary gland development

In recent years, knowledge of fruit fly development has served as a springboard for developmental studies in non-model arthropods like mosquitoes. Embryonic development of mosquitoes is superficially comparable to that of *Drosophila* in that mosquitoes, like fruit flies, are long germ-band holometabolous insects. However, although comparison of *D. melanogaster* and *Ae. aegypti* development suggests that major developmental events are generally well conserved between the two species, the *D. melanogaster* and mosquito insect lineages separated 260 million years ago, suggesting that detailed comparative analyses may uncover divergent developmental processes among these insects [3,11]. This investigation has indeed revealed both divergent and conserved mechanisms of salivary gland development in *D. melanogaster* and *Ae. aegypti.*

The salivary glands of *Ae. aegypti* do not originate from *Drosophila*-like salivary gland placodes located in the VNE, but instead from rudiments located on either side of the proventriculus (Figures 1, 2 and 3). These findings are in agreement with the morphological analyses of *Ae. vexans* development described by Horsfall *et al*. [18]. However, a report on *Culex fatigans* embryology [27] suggests that the aedine mode of salivary gland development may not be found in all mosquitoes. Davis [27], who prepared a brief description of salivary gland development, reported that ventrolateral surface cell division results in a pair of shallow invaginations that are the rudiments of the *C. fatigans* salivary gland. Her description, which is reminiscent of *D. melanogaster* salivary gland development [16,17], suggests that these invaginations deepen and grow posteriorly, eventually lying beneath the stomodeum and the anterior end of the midgut rudiment strands. Likewise, the existence of ventral salivary gland placodes has been reported in the mulberry silkworm *Bombyx mori*[28], suggesting that these structures may be present outside of the Diptera. However, based on their analysis of *Bombyx mori silk gland factor-1*, Kokubo et al. [29] concluded that the *B. mori* silk gland, not its salivary gland, is homologous to the *D. melanogaster* salivary gland. These observations, in conjunction with this investigation, suggest that it will be interesting to assess salivary marker gene expression in additional mosquitoes and other arthropod species.

Although divergent mechanisms for initiation of salivary gland development were observed, many of the genes that regulate salivary gland development in *D. melanogaster* are also expressed in the developing *Ae. aegypti* salivary gland. Thus, despite significant differences between the initiation of salivary gland development in the two species, expression of marker genes, including secretory genes (Figure 3), is conserved. Interestingly, comparable findings have been reported in our previous analyses of arthropod ventral nerve cord development [30-32]. These studies demonstrated that homologous neurons and ladder-like axon tracts were ultimately formed despite differences in the neuroblasts that produced these neurons in various arthropod species, as well as divergent mechanisms for forming the axon tracts in their ventral nerve cords.

### Advancing the study of mosquito development

Although the salivary glands of adult vector mosquitoes have been the subject of many research investigations, this study was the first to assess the genetics of the developing mosquito salivary gland. These studies will promote further investigation of salivary gland development in *Ae. aegypti* as well as other mosquito species. In particular, it may be interesting to assess genes that regulate salivary gland development in the larval, pupal and early adult stages in this species. Such studies may reveal potential targets for intervention.

Future studies may build upon our observation that CrebA is a transcriptional regulator of secretory genes in the salivary gland, as suggested by the identification of CRE sites in multiple secretory pathway genes (Additional file 2), and as functionally assessed through knockdown studies in which CrebA expression was blocked through RNAi (Figure 5). This information may aid in the identification of enhancers that will drive gene expression in the developing salivary gland. For example, the results of this investigation, in conjunction with recent reports describing the introduction of the Gal4/UAS gene misexpression system into vector mosquitoes [33,34], suggest that it may be useful to generate Gal4 strains bearing enhancers with CRE sites. These Gal4 strains could be used to drive misexpression of genes or RNAi constructs in the developing, and perhaps even the adult, salivary gland. Such tools, which would significantly enhance analysis of mosquito salivary gland development, could also be useful in the design of vector control efforts that utilize transgenic mosquitoes.

## Conclusions

This study, the first genetic characterization of vector mosquito salivary gland development, has revealed a set of molecular genetic markers for the developing *Ae. aegypti* embryonic salivary gland. Identification of these markers, which allowed for tracking of salivary gland development, indicated that although many orthologs of *D. melanogaster* salivary gland development genes are expressed in the developing mosquito salivary gland, development of this tissue in *Ae. aegypti* significantly differs from that of *D. melanogaster*. These observations highlighted the need for functional genetic characterization of mosquito salivary gland development, which was explored through RNAi knockdown assays that revealed CrebA as a key regulator of the secretory pathway in the developing *Ae. aegypti* salivary gland. Further analysis of mosquito developmental genetics is necessary and may foster comparative studies of salivary gland development in additional arthropod species.

## Abbreviations

Aae crebA: *Aedes aegypti cyclic-AMP response element binding protein A*; CRE: cAMP response element; siRNA: small interfering RNA; SPCGs: secretory pathway component genes; VNE: ventral neuroectoderm.

## Competing interests

The authors declare that they have no competing interests.

## Authors’ contributions

CN led the marker analysis studies and assisted with characterization of the knockdown phenotypes, figure preparation and drafting of the manuscript. EA performed marker analyses and characterized the knockdown phenotypes. CL assisted with study design and marker analyses. LS performed the knockdown experiments and reared the mosquitoes. ZA prepared the consensus binding site data and assisted with marker analysis. AC assisted with study design, probe preparation and marker analyses; he made the initial observation that initiation of salivary gland development differs in *Ae. aegypti.* DWS assisted with study design and coordination, data analysis and drafting of the manuscript. MDS conceived of the study, prepared probes, and led the study design, coordination and drafting of the manuscript. All authors read and approved the final manuscript.

## Supplementary Material

Additional file 1 ***D. melanogaster *salivary gland marker orthologs in *Ae. aegypti.*** Click here for file

Additional file 2 **CRE sites in the 5′ flanking sequences of *Ae. aegypti *secretory pathway genes.** Click here for file
