# Supplementary material for: Functional genetic characterization of salivary gland development in Aedes aegypti
Source: EvoDevo. 2013 Mar 6;4:9. doi: 10.1186/2041-9139-4-9 (PMC3599648; doi:10.1186/2041-9139-4-9)
Supplement: Additional file 1 — D. melanogaster salivary gland marker orthologs in Ae. aegypti. [file 2041-9139-4-9-S1.pdf]

Associated Gene Name	Description	Dmel Gene ID	Aae Gene ID	Orthology Type
betaCop	coatomer beta subunit	FBgn0008635	AAEL004546	1 to 1
cdc2	cell cycle regulator	FBgn0004106	AAEL008621	1 to 1
crebA	cyclic AMP response element binding protein	FBgn0004396	AAEL000402	1 to 1
da	transcriptional regulator	FBgn0000413	AAEL010226	1 to 1
GlcAT-P	glucuronosyltransferase	FBgn0036144	AAEL006254	1 to 1
Hnf4	transcriptional regulator	FBgn0004914	AAEL011323	1 to 1
l(2)k01209/ucdk1	uridine cytidine kinase	FBgn0022029	AAEL011273	1 to 1
Manf	molecular function unknown	FBgn0027095	AAEL007286	1 to 1
MBD-like	transcriptional regulator	FBgn0027950	AAEL001033	1 to 1
paps	adenylylsulfate kinase; sulfate adenylyltransferase	FBgn0020389	AAEL005605	1 to 1
sage	transcriptional regulator	FBgn0037672	AAEL003613	1 to 1
sar1	COPII component	FBgn0038947	AAEL010012	1 to 1
sec24	COPII component	FBgn0033460	AAEL001273	1 to 1
sec63	ER translocon	FBgn0035771	AAEL007987	1 to 1
sec71	ER translocon	FBgn0028538	AAEL013012	1 to 1
spase22-23	signal peptidase complex component	FBgn0039172	AAEL000947	1 to 1
srp54	signal recognition particle	FBgn0024285	AAEL011568	1 to 1
syx18	SNAP receptor encoding gene	FBgn0039212	AAEL004426	1 to many
TRAM	ER translocon	FBgn0040340	AAEL003285	1 to 1

Additional File 1. *D. melanogaster* salivary gland marker orthologs in *Ae. aegypti*. *Ae. aegypti* orthologs of *D. melanogaster* salivary gland marker genes were identified. The associated gene names, identification numbers in *D. melanogaster* and *Ae. aegypti*, orthology relationships, and gene descriptions (Abrams and Andrew, 2005; McQuilton et al., 2012) are indicated. In this investigation, expression of the *Ae. aegypti* orthologs was assessed during mosquito salivary gland development.
