# Supplementary material for: Functional genetic characterization of salivary gland development in Aedes aegypti
Source: EvoDevo. 2013 Mar 6;4:9. doi: 10.1186/2041-9139-4-9 (PMC3599648; doi:10.1186/2041-9139-4-9)
Supplement: Additional file 2 — CRE sites in the 5′ flanking sequences of Ae. aegypti secretory pathway genes. [file 2041-9139-4-9-S2.pdf]

Additional File 2. CRE sites in the 5' flanking sequences of *Ae. aegypti* secretory pathway genes.

Dmel Gene ID	Aae Gene ID	Orthology Type	Gene Name	Sequences	Strand	Distance to ORF
FBgn0038947	AAEL010012	1 to 1	*sar1*	T T G A C G T T T A	For	500-2000 bp
				T T G A C G T G G A	For	500-2000 bp
				T T G A C G T T T G	For	500-2000 bp
				A G G A C G T T T T	For	500-2000 bp
				T T G A C G T T T C	Rev	<500 bp
FBgn0024285	AAEL011568	1 to 1	*srp54*	T C G A C G T T T T	For	500-2000 bp
				C T T A C G T T G T	Rev	<500 bp
FBgn0028538	AAEL013012	1 to 1	*sec71*	T T G A C G T G T G	Rev	500-2000 bp
				A G G A C G T T A G	Rev	500-2000 bp
FBgn0033460	AAEL001273	1 to 1	*sec24*	G T G A C G T T G C	For	500-2000 bp
				A T T A C G T G T T	For	<200 bp
				A C T A C G T G G A	Rev	500-2000 bp
FBgn0039172	AAEL000947	1 to 1	*spase 22-23*	G C T A C G T T T C	For	<100 bp
				T T G A C G T T T C	Rev	500-2000 bp
FBgn0037672	AAEL003613	1 to 1	*sage*	G C T A C G T T G G	For	500-2000 bp
				A T G A C G T T A A	Rev	500-2000 bp
				T C T A C G T T G C	Rev	500-2000 bp
FBgn0086357	AAEL010716	1 to many	*sec61α*	C G T A C G T T A T	For	500-2000 bp
				T T T A C G T G T A	For	<500 bp
				A C G A C G T T T T	For	<500 bp
				G T G A C G T G A A	Rev	<500 bp
				C T G A C G T T T C	Rev	<500 bp
				T T A C G T T A A G	Rev	500-2000 bp
				C G T A C G T T A C	Rev	500-2000 bp
FBgn0086357	AAEL004523	1 to many	*sec61α*	C C G A C G T T G A	For	500-2000 bp
				A G G A C G T G G C	For	500-2000 bp
				G T G A C G T G A A	Rev	<500 bp
				A T G A C G T G T C	Rev	<500 bp
				T G G A C G T T C C	Rev	<500 bp
				T T T A C G T C T C	Rev	500-2000 bp
FBgn0035827	AAEL015236	1 to many	*srp9*	C T G A C G T T T C	For	<100 bp
FBgn0035827	AAEL012049	1 to many	*srp9*	G A G A C G T T T C	For	500-2000 bp
				T G T A C G T T T C	For	500-2000 bp
				C T G A C G T T T C	Rev	500-2000 bp
FBgn0011509	AAEL009829	1 to 1	*srpRβ*	C G G A C G T A A T	For	<500 bp
FBgn0035947	AAEL010060	1 to 1	*srp68*	T T G A C G T G T T	For	<500 bp
				G C G A C G T G G T	Rev	500-2000 bp
FBgn0037357	AAEL007484	1 to 1	*sec23*	T T G A C G T T C G	Rev	500-2000 bp
				G T G A C G T G T T	Rev	500-2000 bp
				C T G A C G T G T T	Rev	500-2000 bp
FBgn0038808	AAEL001177	1 to 1	*srp14*	T T T A C G T G T C	Rev	<100 bp
				T T G A C G T G C T	Rev	500-2000 bp
				T T G A C G T G C T	Rev	500-2000 bp
FBgn0038810	AAEL003377	1 to 1	*srp72*	C G T A C G T G C A	For	500-2000 bp
				T T G A C G T T G T	Rev	500-2000 bp
				T T G A C G T T T C	Rev	500-2000 bp
FBgn0024509	AAEL012240	1 to 1	*sec13*	T T G A C G T T T A	For	500-2000 bp
				T T G A C G T G G A	For	500-2000 bp
				T T G A C G T T T A	Rev	<500 bp
FBgn0040623	AAEL004603	1 to 1	*spase12*	T T G A C G T T C G	For	500-2000 bp

Dmel Gene ID	Aae Gene ID	Orthology Type	*Gene Name*	Sequences	Strand	Distance to ORF
FBgn0040623	AAEL004603	1 to 1	*spase12*	A T G A C G T T T C	For	<500 bp
				G T G A C G T T T A	Rev	500-2000 bp
FBgn0029709	AAEL000208	1 to many	*CHOp24*	T T G A C G T T T G	Rev	500-2000 bp
FBgn0030306	AAEL003418	1 to 1	*spase25*	G C G A C G T G T C	For	<200 bp
				T T G A C G T T T G	Rev	<500 bp
				G G A C G T G C T A	Rev	<500 bp
FBgn0031049	AAEL005471	1 to many	*sec61γ*	A T G A C G T T G C	For	<100 bp
				C C G A C G T G G A	For	<100 bp
FBgn0010638	AAEL013989	1 to 1	*sec61β*	T T T A C G T T A A	For	500-2000 bp
				C T T A C G T G G T	For	500-2000 bp
				A T T A C G T G G A	Rev	<500 bp
				A T T A C G T G A G	Rev	500-2000 bp
FBgn0010391	AAEL005856	1 to 1	*Gtp-bp*	C T G A C G T T A T	For	500-2000 bp
				G T G A C G T T T A	For	<500 bp
FBgn0011584	AAEL010538	1 to many	*Trp1*	T T G A C G T T T T	For	500-2000 bp
				G G T A C G T T T T	Rev	<500 bp
FBgn0011584	AAEL013226	1 to many	*Trp1*	T T T A C G T T T A	Rev	<500 bp
FBgn0015298	AAEL004914	1 to 1	*srp19*	T T G A C G T T A T	For	<500 bp
FBgn0021795	AAEL013320	1 to 1	*Tapδ*	T G G A C G T G G C	For	<100 bp
				T T G A C G T T C G	Rev	<100 bp
FBgn0022268	AAEL008434	1 to many	*KdelR*	C T G A C G T G T A	For	500-2000 bp
FBgn0022268	AAEL002086	1 to many	*KdelR*	C T G A C G T G T A	For	500-2000 bp
				C T G A C G T G T G	For	500-2000 bp
				T T G A C G T T T G	Rev	500-2000 bp
				T T T A C G T G G C	Rev	500-2000 bp
FBgn0028969	AAEL013230	1 to 1	*δCOP*	T C G A C G T G A T	For	500-2000 bp
				C T G A C G T G T G	Rev	<100 bp
				T T G A C G T T T C	Rev	500-2000 bp
				G T G A C G T T T G	Rev	<100 bp
				A C G A C G T G T G	Rev	500-2000 bp
FBgn0030341	AAEL011755	1 to 1	*p24-1*	T T G A C G T T T G	For	<500 bp
FBgn0040340	AAEL003285	1 to 1	*TRAM*	G G G A C G T A T G	For	500-2000 bp
				A G A A C G T G T T	Rev	500-2000 bp
FBgn0040512	AAEL013121	1 to 1	*ζCOP*	C G G A C G T G A A	For	500-2000 bp
				T T G A C G T T A A	Rev	500-2000 bp
				A T G A C G T T T C	Rev	500-2000 bp
FBgn0053105	AAEL000410	1 to many	*p24-2*	C C G A C G T G T T	Rev	500-2000 bp
				T C T A C G T G T G	Rev	500-2000 bp
FBgn0033339	AAEL001516	1 to 1	*sec31*	A T T A C G T T C C	For	<500 bp
FBgn0010348	AAEL007065	1 to 1	*arf79F*	T C T A C G T G G G	For	500-2000 bp
				G T T A C G T T G C	Rev	<200 bp
				G C T A C G T T T G	Rev	<500 bp
FBgn0035771	AAEL007987	1 to 1	*sec63*	G C G A C G T T T A	Rev	<200 bp

Ae. aegypti orthologs of *D. melanogaster* secretory genes were identified. The associated gene identification numbers, orthology relationships, and gene names are provided. A 2 kb region immediately upstream of the open reading frame (ORF) of each *Ae. aegypti* secretory pathway gene ortholog was searched for sequences corresponding to the CRE-binding site consensus motif (Abrams and Andrew, 2005). The identified sequences and their locations with respect to the open reading frame (ORF) of each *Ae. aegypti* gene are indicated.
